# Toward ethical human microbiome research: improving health through radical interdisciplinary and intercultural co-laboration

**DOI:** 10.1186/s40168-026-02340-w

**Published:** 2026-04-07

**Authors:** Wim Van Daele, Raul Yhossef Tito Tadeo, Jennifer Perera, Tshokey Tshokey, Per Ole Iversen, Saroj Jayasinghe, Roberta Raffaetà, Neyzang Wangmo, Heidi E. Fjeld, Sonam Chhoden R, Tharanga Thoradeniya, Jeroen Raes

**Affiliations:** 1 Department of Nutrition and Public Health, University of Agder, PO Box 422, 4604 Kristiansand, Norway; 2 Department of Microbiology and Immunology, Rega Institute, KULeuven, Leuven, Belgium; 3 Center for Microbiology, VIB, Leuven, Belgium; 4 Department of Medical Microbiology and Immunology, University of Colombo, No.25, Kynsey Road, Colombo – 08, Sri Lanka; 5 Department of Pathology and Laboratory Medicine, Jigme Dorji Wangchuck National Referral Hospital, PO Box No. 128, Thimphu, Bhutan; 6 Department of Nutrition, University of Oslo, PO Box 1046, 0317 Oslo, Norway; 7 Department of Haematology, Oslo University Hospital, Oslo, Norway; 8 Division of Human Nutrition, Stellenbosch University, Tygerberg, South Africa; 9 Department of Clinical Medicine, University of Colombo, Colombo – 08, Sri Lanka; 10 Department of Philosophy and Cultural Heritage, Ca’Foscari University of Venice, Venice, Italy; 11 The New Institute: Center for Environmental Humanities, Cà Foscari University of Venice, Venice, Italy; 12 The Medical Education Centre for Research Innovation and Training (MECRIT), Khesar Gyalpo University of Medical Sciences Bhutan, Menkhang Lam 3, Thimphu, Bhutan; 13 Department of Community Medicine and Global Health, Institute of Health and Society, University of Oslo, P.O box 1130, 0318 Oslo, Norway; 14 Faculty of Undergraduate Medicine, Khesar Gyalpo University of Medical Sciences of Bhutan, Menkhang Lam 3, Thimphu, Bhutan; 15 Department of Biochemistry and Molecular Biology, Faculty of Medicine, University of Colombo, No.25, Kynsey Road, Colombo–08, Sri Lanka

**Keywords:** Ethics, Inclusivity, Co-laboration, Radical interdisciplinarity, Microbiome, Co-laborative science, Interculturality, Decolonization, Global health

## Abstract

As human microbiome research is globalizing, it raises ethical concerns regarding the European and North American dominance in the field, which may reproduce a colonial bias and perpetuate inequities in global health research and outcomes. We suggest disentangling this ethical quandary into three main concerns: 1) scientific bias toward European and North American populations; 2) limited meaningful community inclusion, participation, and ownership, and 3) scant significant inclusion of diverse global researchers. We then formulate three recommendations for their resolution, deploying *co-laboration*—joint *labor* of diverse partners in generating synergies between diverse disciplines, cultures, and knowledges around shared concerns—and *co-laborative science*—a form of citizen science based on such synergies between diverse partners—to guide meaningful inclusive, participatory, and ethical human microbiome research. To conclude, we promote a programmatic list for putting co-laborative ethical science into practice, benefiting global communities, individuals, and researchers alike and decolonizing and improving health worldwide.

## Ethics at the center of human microbiome research

Human and non-human microbiome studies are flourishing. Unprecedented strides in understanding host-associated microbial variation have been made, with many clinical opportunities on the horizon [1, 2]. Meanwhile, the field is globalizing and entering novel domains, populations, and regions, raising ethical concerns regarding the European and North American (excluding Mesoamerica) dominance in the field. This imbalance in microbiome and, more broadly, global health research has grown from historical colonial injustices and constitutes a profound ethical problem as it may contribute to perpetuating profound inequities in health research and outcomes, ultimately concerning the uneven distribution of life and death [3, 4]. Hence, we join the growing call for the decolonization of microbiome and global health research and science in general [5–13].

As part of this call, *Nature* published a new framework in June 2022 to counter “helicopter research” in which privileged and well-funded scientists from the Global North descend from highly-equipped laboratories to collect samples from ‘exotic’ people and extract the material for analyses in inaccessible high-tech laboratories for the sole benefit of European and North American societies. *Nature*’s framework instead seeks to enhance inclusion and strengthen ethics in global collaborations [14], a concern reiterated more recently [7], and stresses the urgent need to expand the consideration of harm from individuals to entire populations [15]. Similarly, *The Lancet* commits to advancing equity, diversity, and inclusion (https://www.thelancet.com/equity-diversity-inclusion), refusing for instance, articles concerning African data that do not acknowledge the African collaborators upon which they depend (https://www.universityworldnews.com/post.php?story=20220603115640789); this inequality is further addressed in The Cape Town Statement on fairness, equity, and diversity in research [16] and The Africa Charter (https://parc.bristol.ac.uk/africa-charter/). Applied to microbiome research, a recent paper [17] proposes a list of concrete suggestions for ethical research, further elaborated in other publications [8, 18–20]; this mirrors issues raised in the social sciences [21, 22] having spurred novel initiatives, such as the Microbes and Social Equity Working Group [23]. These growing calls have contributed to the inclusion of health equity in the updated Declaration of Helsinki stressing that inclusive health research is essential in research ethics [24].

These calls and initiatives recognize and seek to address the overall European and North American dominance in setting the research agenda, biasing scientific knowledge, and perpetuating global inequalities in microbiome and health research and an unfair distribution of clinical applications across different populations, which constitutes a fundamental ethical quandary.

## Disentangling the ethical knot

We discern three concerns marked by a fundamental lack of diversity: 1) bias toward European and North American populations in human microbiome research; 2) limited meaningful community inclusion, participation, and benefit sharing; and 3) scant significant inclusion of diverse global researchers in current collaborations.

1. European and North American bias in current microbiome research
  A recent metastudy revealed that 71% of microbiome samples of known origin are collected from Europe, the US and Canada, with 46.8% alone from the US, which contains only 4.3% of the world’s population. In contrast, India, Bangladesh, and Pakistan account for more than 26% of the world’s population, but only 1.8% of microbiome samples [25, 26]. Even though China constitutes a global exception to the rule and an increasing amount of samples are collected across the world, the high proportion of samples collected among European and North American populations remains constant and studies of microbiome-disease associations continue to focus on these regions’ health conditions that define the research agenda globally [19, 27]. As studies on non-Western populations expose considerable variation in the gut microbiome in general and in relation to ways of living (including food patterns), host health and disease, and microbiome-affecting interactions with varied environments and animals [20, 27–36], this bias may distort the very science of the human microbiome and its role in health and disease, and may obfuscate its global diversity. This quandary was raised over a decade ago and is reiterated with regard to other health-related fields, such as genomics [37]. The bias then impairs an understanding of global variations in microbiome-host-health-environment interactions and may then predominantly cater to European and North American populations due to limited transferability or generalizability of findings across different populations and geographies [20]. Hence, the risk exists that a biased microbiome science will fail as a global standard for diagnosis, clinical microbiome testing, devising effective therapies and prevention strategies for these other populations [26, 38, 39] risking that clinical programs must be redone or, worse, never done because of limited interest; this perpetuates exclusion and inequality that boils down to those setting, defining, and funding the agenda.
2. Limited meaningful community inclusion, participation, and benefit sharing
  Even when research is conducted on populations other than Europeans or North Americans, it is also not without its challenges. This is epitomized in “helicopter research” where disadvantaged communities may easily lose control over this process as they are insufficiently involved in all phases from planning the research to sharing the benefits [8, 14, 18]. Hence, we applaud the growing call for increased participation by Indigenous communities [8, 18, 29, 40], but stress the need to expand this to include populations in the Global South, as part of an overall decolonization of global health [5, 6, 12], and other groups who face multiple dynamics of marginalization which calls for an intersectional approach in which superposing exclusionary dynamics are carefully addressed (e.g., Afro-American lesbian women) [41]. Yet, we must be explicit about the kind of participation that is promoted and what it entails [42], as there are many different degrees — from delivering data to shaping authoritative knowledge — and forms — community-based participatory research, participatory action research, citizen science, and more — of participation [41–48]. The degree, form and context of participation by heterogenous populations influence whether or not they experience it as meaningful [42, 49]; this hinges also on the degree of recognition and inclusion of their different knowledges or epistemologies in the process of scientific knowledge production [8, 50, 51]. A lack of true inclusion of these populations in research hampers meaningful participation and prevents them from becoming co-authors in the production of scientific knowledge (and thus placing items on the research agenda) that lays the ground for shared benefits which are deemed meaningful for the communities.
3. Scant significant inclusion of diverse researchers in current collaborations
  There is a rampant inequality in global collaborations in many disciplines, not only in microbiome research; this expresses itself foremost in issues around funding distribution, acknowledgement of credit and authorship, and sharing of benefits [19, 52, 53]. A recent metastudy [54] has shown that 79.8% of the articles on African microbiomes have neither a first nor last author connected to an African institution, with 43.5% of first authors affiliated to a US institution. One explanation for this lack of African leadership in studies of African microbiomes lies in the inequality of funding resources; American scholars have these resources and thereby become the Principal Investigators. This situation turns economic inequality into scientific and reputational inequality [54], which then becomes economically perpetuated in funding streams favoring highly reputed researchers. The varied contexts in which these African and other scholars and their institutions from the global South find themselves (e.g., strong or poor funding environment) structurally perpetuate historically shaped relations between scientific collaborators and institutions in international teams as well as emerging research agendas and thus bias in science [20]. This also includes the issue of epistemic injustice in which different knowledges situated in the global South are often excluded or relegated to secondary roles [12]. Such structural inequity is an ethical problem, since it does not provide due recognition to the work and contributions made by these scholars and collaborators (and participants) upon which the success of the European and North American investigators depends and also prevents scientists and scholars in the South from developing their own research agenda, knowledges, successes, and reputation [8, 22, 23].

## Recommendations for solving ethical challenges

To respond to these ethical quandaries and distinguish ourselves from problematic forms of collaboration, we adopt and elaborate the notion of *co-laboration* [55–57], stressing the need for radical interdisciplinarity and interculturality in order to de-bias and diversify microbiome science and render it more inclusive and participatory. Radical refers here to profoundly engaging with different scientific and alter-native worldviews and knowledge practices. Co-laboration entails experimentation in working with various partners and experts, not only scientists and academics, and their knowledges or epistemologies around shared concerns pertaining to microbes and health and disease to create synergies [12, 56] (https://easst.net/easst-review/382/laboratory-anthropology-of-environmenthuman-relations/). The term was chosen to highlight the laborious, experimental, co-creative nature of this approach. Co-laboration requires time and effort, where frictions may surface; these are acknowledged and openly dealt with. The microbiome as a shared matter of concern then involves the human sciences — anthropology, sociology, political sciences, history — and microbiological sciences, as well as concerned community stakeholders jointly shaping microbiome research from inception to translation into therapies and medicines. Even though it may be an arduous and time-intensive process, it is worthwhile for increasing the generalizability of microbiome science and rendering it more ethical by addressing globally and locally situated environment-host-microbiome-disease interactions to improve health across different populations. In line with this, we also propose a model of *co-laborative science* in which different partners, disciplines, and knowledges (e.g., Indigenous knowledge) *co-labor* toward each other in creating synergies, mutually affecting and enriching each other around shared concerns, to create a science attentive and adaptive to different socio-ecological environments, worldviews, and related practices shaping microbiomes, health and diseases of different populations. It is a particular form of citizen science that transcends disciplinary frames, meaningfully includes non-academic stakeholders, and guides truly intercultural, decolonial and inclusive ethical research practice. The way this overall approach plays out in our three recommendations is outlined below:

1. Deconstructing the current bias
  To de-bias microbiome research and increase our sensitivity to the human variation to which therapies and prevention programs should be adjusted, we first need to conduct more microbiome studies across various global communities, while avoiding the pitfalls of extractivism [8, 20, 22, 27, 58, 59]. We also still need more research on the influences of geographical location, cultural praxis, socio-economic status, food and dietary intake, use of antibiotics, and other “environmental” factors on the microbiome. This will provide a more solid research basis for reconsidering some of the empirically and ethically problematic analytical categories often deployed in microbiome research among understudied populations (e.g., traditional vs. modern) [13, 20, 29, 30, 60–67]. Ethical microbiome research requires careful development and deployment of analytical and conceptual categorizations, and these should also exhibit a detailed sensitivity towards the empirical complexity of the contexts in which different populations live. For instance, most Indigenous people are not living in an ancient pristine past as they are likewise affected by historical contacts, pollution, climate change and globalizing food systems [13, 19, 65]. Understanding these contextual processes shaping human and microbial life will aid in avoiding such neo-colonial frameworks of analysis, diversifying microbiome research, and situating the microbiome in its socio-ecological environments. Such situated and environmentally sensitive microbiome research can even empower indigenous and disadvantaged groups in rectifying and addressing historically and socially patterned uneven exposures to detrimental ecological and biological influences affecting the gut microbiome and ultimately health yet provided a careful deployment of analytical terminology [68]. Developing such sensitive, contextual and situated understanding requires co-laboration with nutritionist, health experts, anthropologists, social scientists, and local scientists and interlocutors who should be introduced to each other’s fields and knowledges to enable true synergies. Importantly, it is not sufficient to include people only physically located in the Global South, but it also requires having partners situated within knowledge systems from the Global South [12]. These scholars and interlocutors are also well-placed for interculturally brokering of relations of mutual understanding [10] in the inter-knowledge efforts of co-laborative science to enable true participation by concerned partners and communities. As such, co-laborative microbiome research becomes more empirically accurate, pluralistic, inclusive, non-stigmatizing, and ethical.
2. Establishing meaningful community inclusion, participation, and benefit sharing
  With growing calls for decolonization, it is crucial to address the ethical issues of partnership, participation, and ownership by developing a sustained dialogue with the communities involved. Meaningful participation entails involving these communities and their expertise and knowledges from the start when setting the agenda — crafting research questions, developing research designs, acknowledging contributions, benefit sharing — so that they can participate fully in authoritative knowledge production. Such engagement requires efforts from all sides and requires building long-term partnerships to develop a synergy between partners and knowledges, and iteratively strengthen meaningful participation and trust over time [69]. Stressing the need for inclusiveness of different knowledge systems [12, 20, 51, 70] and solid participation throughout in the co-creation of scientific knowledge, we propose the model of co-laborative science as an alternative form of citizen science; this avoids association with some of the latter’s neocolonial and exclusivist forms where only scientific knowledge is deemed superior and where other knowledge systems are relegated to secondary roles. Our proposed ethically and culturally sensitive approach requires an enduring engagement, but allows the building of meaningful long-term partnerships, trust, empowerment and innovative science [8, 32]. We also propose co-laborative science as an alternative model of citizen science to avoid the latter’s associations with citizen and recognition discourses [50]; these discourses may have exclusionary associations for certain communities, such as those facing exclusion from citizenship (e.g., migrants) and authoritarian states granting few rights [41, 46]. Several alternative terminologies [44–46, 71] have been proposed to avoid these potential negative connotations and exclusionary effects of citizen science on different communities, but we deem these as insufficiently globally applicable; we surmise that our proposed co-laborative science fulfills this more generalizable ambition while remaining flexible to local adaptation. Co-laboration emphasizes intercultural and inter-knowledge brokerage in joint authoritative knowledge production; through this, participation becomes truly meaningful, and ownership is enhanced. It also involves experimentation, entailing trial and error, which is hinted at by the co-laborative science model through its association with the term laboratory. We surmise that our proposed model, when applied and adapted across the world, including among disenfranchised groups [45, 46, 48], can avoid stigmatizing categorizations and propel inclusive, ethical, and truly participatory research practice on human microbiomes.
3. Developing mutually enriching global (scientific) co-laborations
  To address the historical structural inequalities shaping some global collaborations, we propose co-laboration, entailing interdisciplinary and intercultural global teams as well as inter-knowledge and intercultural brokerage. Co-laborative science includes microbiologists, anthropologists, nutrition and global health experts, as well as other scholars from relevant geographical locations and knowledge systems, and stakeholders from the concerned communities; it involves mutual capacity-building and learning in the process, but transcends it by aiming for local leadership in microbiome research. Specific intersectional consideration is required to include scholars who face multiple dynamics of exclusion. To achieve co-laborative science, we need appropriate funding streams and structures to create spaces for radical interdisciplinarity, inclusion of diversified knowledges, and the creation of infrastructure and space to boost decentralized scientific creativity and ownership, enriching the global scientific project of the human microbiome.

By adopting and adapting co-laboration and co-laborative science we wish to emphasize the mutual work required to achieve such radical forms of participation and inclusion. It requires establishing a solid understanding of the context and socio-ecological environments in which the concerned populations live as well as co-elaborating how partnerships and participation should take shape according to the specific situation and context. This is a gradual process that should start rather sooner than later, as it concerns life and health and their fair distribution [4]. Several of us are currently working towards and edging closer to the goal set out in this correspondence, some of which published [69]. Throughout several projects, we continue to learn iteratively through our partners on how to further improve while we spend considerable and worthwhile time building such partnerships, obtaining more funding, and implementing co-laboration in diverse settings. Hence, our proposed way of working aims at promoting an empirically sound and situated approach to microbiome studies across the world that strengthens ethical research practices (Table 1).

**Table 1:** Central elements of collaboration vs. co-laboration (upper panel) and citizen science vs. co-laborative science (lower panel)

Collaboration - A container concept involving highly varied forms - Different degrees and forms of participation - Different constellations of power relations between knowledges	Co-laboration - A type of collaboration entailing radical participation - Acknowledging power constellations and being prepared to work toward symmetrical power relations - Radical interdisciplinarity and interculturality - Experimentation with trial and error - Laboring toward mutual enrichment
Citizen science - Different forms, some of which regard scientific knowledge as *the* standard - Different degrees of participation in authoritative knowledge production - Associated with citizen discourse, with potential exclusionary connotations for certain communities	Co-laborative science - Alternative model of citizen science - Inclusive of different knowledges - Meaningful and sustained participation in authoritative knowledge production - Inclusive co-creation and experimentation from inception to publication - Radical interdisciplinarity and interculturality - Creating decentralized scientific activity in a multipolar world

## Strategies for achieving radical interdisciplinary and intercultural co-laboration for ethical research practice

Here, we propose a programmatic agenda for radical interdisciplinary, intercultural, and ethical co-laborative microbiome research.

1. Debias and diversify microbiome science
  - Allocate funding for diversifying research on non-European and non-Euro-American microbiomes, avoiding the pitfalls of extractivism and helicopter research, setting aside funding for developing long-term relationships and co-creating knowledge.
  - Deploy social scientists, such as social anthropologists, and community interlocutors to understand the situated context and the socio-ecological processes shaping and being shaped by microbiological circulations in the concerned population’s life and to identify potential partners for setting up the research team. Depending on the local situation, it may be necessary to broker a prior intercultural and meaningful communication about microbiomes between scientists and identified stakeholders from the community, especially where communities are unfamiliar with the concept of microbes.
  - Establish a radical interdisciplinary and intercultural global team, comprising (micro)biological, global health experts and social scientists as well as stakeholders. Prepare sufficient time for thorough communication so that all parties have a sufficient understanding of the sciences, global and local dynamics, knowledges, and concepts, since similar concepts may have different meanings cross-disciplinarily and cross-culturally. This process may be started in ongoing projects to iteratively strengthen communication for future co-laborative programs in the long run.
2. Establish a program for meaningful co-laborative science (with inclusion of non-academic partners)
  - Design a shared research agenda with all scientists, stakeholders, and concerned populations from inception to execution of the research, leading to mutually enriching knowledge, ongoing co-creation of the research, and authoritative knowledge.
  - Establish a meaningful informed consent process with communities, groups, and individuals; this would not be a one-off event but would take place during different research phases and research would be modified accordingly and iteratively. As informed consent presupposes that one understands what one is consenting to, it is necessary to train local scientists and community leaders and organize multiple information sessions so that local partners and communities can assess what they consider appropriate and if and how they wish to contribute to and shape the research (a process initiated in the first set of strategies). During this information process, inter-knowledge and intercultural brokerage (by either anthropologists or Indigenous scientists) are essential to avoid neo-colonial approaches in the encounter [72] and create a truly participatory agenda setting [73].
  - Make inclusiveness as well as inter-knowledge and intercultural brokerage the central tenets of the co-laborative science and develop these iteratively throughout the entire process, maintaining a meaningful partnership among all stakeholders throughout the research program where potential frictions can be acknowledged and resolved.
  - Establish a plan together as partners from the start, acknowledging everyone’s long- and short-term contributions throughout the concerted labor of co-creation, discussing the equitable sharing of outputs and benefits, enhancing ownership and empowerment. This includes explicit discussions regarding the ownership of biological material, criteria of compensation, sharing of intellectual property rights, acknowledgement of contributions, etc. This may need to be readjusted jointly along the way.
  - Set up a plan for distribution of funds towards disenfranchised partners to enable radical interdisciplinary and intercultural co-laboration and ownership by all partners — local scientists, leaders, and communities — in the project, so they can gradually take matters into their own hands and have more power to determine their research agendas; capacity-building is an important yet insufficient part of this.
  - Develop radical interdisciplinary/transdisciplinary (e.g., microbiosocial) conceptual toolboxes and meta-level theories that allow for a radical integration and representation of different knowledges held by diverse stakeholders; these aid in further developing innovative research designs and projects that are radically equitable, culture-sensitive, and ethical.
  - Co-create knowledge through co-laboration throughout the research cycle, including sharing findings and conclusions, discussing frictions, and being open to changing position iteratively, and sharing benefits with the involved scientists, stakeholders, and concerned populations.
3. Develop meaningful global (scientific) co-laborations
  - Ensure that everyone’s contributions are fully acknowledged throughout so that reputation and expertise can be built and strengthened globally.
  - Establish a sustained partnership and devise a long-term plan for securing funding. As such, research groups in the Global South can acquire the structural conditions to decide on the research agenda and further co-laborations as well as develop independent research cultures. Such includes, besides establishing funding structures, developing pooled funding models, sharing of infrastructure, transferring technologies and knowledge, aiding in publishing open access, and the like. Engaging in such co-laborative partnerships will be more sustainable and ethical in a decolonizing and multipolar world.
  - Assist in the creation of decentralized and creative scientific research cultures and ownership; this includes capacity-building but requires all the above.

Adopting the above concrete strategies to achieve ethical co-laborative microbiome and decolonial global health science will require scholars to think out of the (disciplinary) box, assess one’s approach and knowledge critically, restructure funding streams, and move outside the academic comfort zone. These strategies are presented as a proposal to strengthen the very knowledge of microbiomes across different populations and settings, and ethical microbiome research practices in global contexts. We are well aware that each context is different, and this will require adjusting the strategies accordingly. Yet, strengthening meaningful participation and inclusion in globalizing microbiome research is in itself an ethical obligation as it concerns ultimately the fair distribution of health and life, and it is no surprise therefore that the Helsinki Declaration has included health equity as a key pillar in ethical research practice [24]. This demands a concerted and sustained effort and focus, to which we hope we have contributed here (Table 2).

**Table 2:** Overview of the arguments

Ethical challenges	Broad recommendations	Programmatic strategies
• Bias toward European and Euro-American populations • Limited meaningful community inclusion, participation and benefit sharing • Scant significant inclusion of diverse researchers in global scientific collaborations	• Deconstruct the bias in microbiome science • Establish community inclusion, meaningful participation and benefit sharing • Develop mutually enriching global (scientific) co-laborations • All three require radical interdisciplinary and intercultural co-laboration in the model of co-laborative science which serve as alternatives to some of the problematic collaboration and citizen science models	• For debiasing the Euro-American microbiome: Establish inclusive teams and research agenda's • For community inclusion, participation and benefit sharing: Planning the research together from inception to sharing of benefits, which includes interknowledge and intercultural brokerage • For inclusive global co-laborations: Develop partnerships with the aim of creating independent research cultures

## Data Availability

No datasets were generated or analysed during the current study.
